# High intensity interval training vs. moderate intensity continuous training on aerobic capacity and functional capacity in patients with heart failure: a systematic review and meta-analysis

**DOI:** 10.3389/fcvm.2024.1302109

**Published:** 2024-02-21

**Authors:** Changran Yang, Lizhuang Zhang, Yu Cheng, Manman Zhang, Yuxin Zhao, Tianzi Zhang, Jiawang Dong, Jun Xing, Yuzhi Zhen, Cuihua Wang

**Affiliations:** ^1^ Department of Rehabilitation, The First Hospital of Hebei Medical University, Shijiazhuang, Hebei, China; ^2^ Department of Cardiology, The First Hospital of Hebei Medical University, Shijiazhuang, Hebei, China

**Keywords:** heart failure with reduced ejection fraction, high-intensity interval training, moderate-intensity continuous training, exercise capacity, functional capacity

## Abstract

**Background:**

Exercise training is commonly employed as a efficacious supplementary treatment for individuals suffering from heart failure, but the optimal exercise regimen is still controversial. The objective of the review was to compare the effects of high-intensity interval training (HIIT) and moderate-intensity continuous training (MICT) on the exercise capacity, cardiac function, quality of life (QoL) and heart rate among patients with heart failure with reduced ejection fraction.

**Methods:**

A systematic search was performed using the following eight databases from their inception to July 5, 2023: PubMed, Web of Science, Embase, Cochrane Library, Clinical Trials, China Knowledge Network, Wan fang Data, and the China Biology Medicine databases. The meta-analysis results were presented as mean difference (MD) and 95% confidence interval (CI). The Cochrane Risk of Bias tool was used for the included studies. The Grading of Recommendations Assessment, Development, and Evaluations was used to assess the certainty of evidence.

**Results:**

Thirteen randomized controlled trials were included in the study. The results showed that HIIT had a significant positive effect on peak oxygen uptake (MD = 1.78, 95% CI for 0.80–2.76), left ventricular ejection fraction (MD = 3.13, 95% CI for 1.25–5.02), six-minute walk test (MD = 28.13, 95% CI for 14.56–41.70), and Minnesota Living with Heart Failure Questionnaire (MD = −4.45, 95% CI for −6.25 to −2.64) compared to MICT. However, there were no statistically significant differences observed in resting heart rate and peak heart rate.

**Conclusions:**

HIIT significantly improves peak oxygen uptake, left ventricular ejection fraction, six-minute walk test, and Minnesota Living with Heart Failure Questionnaire in patients with heart failure with reduced ejection fraction. Additionally, HIIT exhibits greater effectiveness in improving peak oxygen uptake among patients with lower body mass index.

**Systematic Review Registration:**

https://www.doi.org/10.37766/inplasy2023.7.0100, identifier (INPLASY2023.7.0100).

## 1 Introduction

Heart failure (HF) is characterized by typical signs and symptoms due to structural and/or functional cardiac abnormalities. It is commonly characterized by dyspnea, fatigue, and leg swelling, which may be accompanied by peripheral edema and elevated jugular venous pressure (1). The 2016 guidelines of the European Society of Cardiology classify left ventricular ejection fraction (LVEF) ≤ 40% as HF with reduced ejection fraction (HFrEF) (2). Reduction in exercise tolerance has been suggested to predict readmission and mortality in HF patients (3). In 1990, Coats et al. conducted the first study on HF and exercise training, which demonstrated that patients with HF benefit from exercise training by improving their aerobic capacity and physical fitness (4). Exercise training has become a common adjunctive treatment for individuals suffering from HF, as recommended by the American College of Cardiology (ACC) at level 1 (5). Furthermore, Antunes et al. (6, 7) demonstrated that exercise training improved functional capacity, exercise endurance, and quality of life (QoL) for patients with HFrEF.

Among the various training methods utilized for individuals with cardiovascular disease, moderate-intensity continuous training (MICT) is the most frequently used, with an intensity of 50%–60% peak oxygen uptake (Peak VO_2_) or 50%–75% peak heart rate (HR peak) (6). MICT is characterized by lower intensity, longer duration, and a higher level of safety (7). Recent studies (8, 9) have indicated that high-intensity interval training (HIIT) has the potential to greatly enhance the aerobic capacity of individuals with HF and coronary artery disease. HIIT involves short bursts of intense exercise lasting between 10 s and 5 min, during which individuals reach 80%–90% of their Peak VO_2_ or 85%–95% of their peak heart rate. These high-intensity periods are interspersed with intervals of low-intensity exercise at less than 45% of Peak VO_2_ or less than 80% of peak heart rate, or rest (6). HIIT exhibits diversity in its types, incorporates a specific recovery interval, and achieves comparable training effects to MICT within a shorter duration. Additionally, HIIT elicits an intense cardiovascular stimulation and enhances the enjoyment and concentration of exercise (10, 11).

A meta-analysis comparing HIIT and MICT for HF indicated that HIIT yielded superior improvements in Peak VO_2_ compared to MICT, although no significant difference was observed in the enhancement of QoL (12). Tucker et al. concluded that HIIT was superior to conventional exercise training but not MICT in terms of improving left ventricular ejection fraction (LVEF) and Peak VO_2_ (13). Heart failure with reduced ejection fraction (HFrEF) is a syndrome characterized by high mortality and morbidity (14). However, there are no sufficient and conclusive studies regarding the impact of HIIT on HFrEF. Furthermore, the majority of published studies comparing HIIT and MICT in HF have primarily focused on heart failure with preserved ejection fraction (HFpEF). Therefore, it is necessary to clarify the clinical effects of HIIT specifically in HFrEF (15–17). Furthermore, it is crucial to consider energy expenditure during training when comparing various exercise regimens. The aim of isocaloric protocols is to equalize energy expenditure during aerobic exercises of varying intensities. Additionally, the enhancement of exercise capacity in individuals with HF is primarily influenced by energy expenditure (18). A previous study by Mansueto et al. discovered that HIIT was superior to MICT in improving Peak VO_2_, the superiority disappeared in subgroup analyses based on isocaloric protocols, however (19).

Consequently, in order to provide more comprehensive information and guidance for clinical practice, in the meta-analysis, the effects of HIIT and MICT on aerobic exercise capacity, cardiac function, QoL, and heart rate among patients with HFrEF were compared, based on previously conducted randomized controlled trials. Moreover, a sub-analysis of isocaloric exercise training studies is also included in the systematic review.

## 2 Materials and methods

This systematic review and meta-analysis were conducted and reported in accordance with the Preferred Reporting Items for Systematic Reviews and Meta-Analyses (PRISMA).

### 2.1 Data source and search strategy

A comprehensive literature search was conducted using the following eight databases from their inception to July 5, 2023: PubMed, Web of Science, Embase, Cochrane Library, Clinical Trials, China Knowledge Network, Wan fang Data, and the China Biology Medicine databases. The following keywords were used as the search terms: “Heart Failure/Cardiac Failure/Heart Decompensation”; “High-Intensity Interval Training/High-Intensity Intermittent Exercise/HIIT/Sprint Interval Training” and their related terms. In addition, all reference lists of eligible studies were reviewed to identify any additional studies that could be included. There are no language restrictions on the search strategy, which is shown in Supplementary Table S1. The systematic review protocol was registered on INPLASY (INPLASY2023.7.0100) and is available on the website inplasy.com (https://www.doi.org/10.37766/inplasy2023.7.0100).

### 2.2 Inclusion and exclusion criteria

Following the removal of duplicate studies, titles and abstracts of the remaining articles were assessed, and subsequently, their full texts were examined to identify potentially eligible studies. Studies were selected based on the following criteria: (1) Randomized controlled trials (RCTs) involving adult individuals (≥18 years) diagnosed with HF with reduced ejection fraction (LVEF ≤40%); (2) HIIT in the intervention group vs. MICT in the control group; (3) The exercise intensity in both groups met the inclusion criteria established by Weston et al. (20); (4) The outcome measures included at least Peak VO_2_. Exclusion criteria were: (1) patients with HF with preserved ejection fraction (LVEF >50%) or those with unreported LVEF; (2) incomplete articles, non-randomized controlled trials; (3) not MICT as the control group; (4) missing original data, reviews, meta-analyses, and animal experiments. The primary outcome measure for this review was Peak VO_2_, and secondary outcomes included six-minute walk test (6MWT), left ventricular ejection fraction (LVEF), Minnesota Living with Heart Failure Questionnaire (MLHFQ), resting heart rate (HR rest), and peak heart rate.

### 2.3 Data extraction and quality assessment

Based on studies that met the inclusion criteria, the following information was extracted: (1) authors of the study and publication year; (2) the mean age, gender, and body mass index (BMI) of the participants; (3) sample size; (4) frequency, intensity, time, and modality of training in the HIIT and MICT groups; (5) mean and standard deviation of the outcome measures. To obtain the original data, we will contact the authors if complete data are not provided in the publication.The risk of bias was assessed by following the Cochrane guidelines for RCTs in several areas: (1) the generation of sequences and allocation concealment; (2) blinding of participants and personnel; (3) blinding of outcome assessment; (4) incomplete outcome data; (5) selective reporting and other potential biases. According to established criteria, the risk of bias was classified as low, uncertain, or high.

### 2.4 Quality of meta-analysis evidence

Grading of Recommendations Assessment, Development and Evaluation (GRADE) guidelines were used to assess the quality of evidence for the outcomes. The evaluation included the assessment of five GRADE items: (1) bias risk, (2) inconsistency, (3) indirectness, (4) imprecision, and (5) publication bias (21). There were four levels of certainty of evidence: high, moderate, low, and very low.

### 2.5 Data synthesis

The mean differences (MD) and standard deviations from baseline to endpoint were extracted and included in the database for analysis for each group. The mean difference (MD) and 95% confidence intervals (95% CIs) were utilized for result comparison, but when outcomes were measured in the different ways, the standardized mean difference (SMD) and 95% CIs were used. The analysis was performed using Rev Man 5.3, and heterogeneity was assessed using the *I*^2^ statistic. *I*^2^ values of 25%, 50%, and 75% indicated low, moderate, and high levels of heterogeneity, respectively (22). If moderate or high heterogeneity (*I*^2^ > 50%) was detected, a random-effects model was applied; otherwise, the fixed-effects model was employed. Statistical significance was determined at *p* < 0.05, and the effect sizes and 95% confidence intervals were graphically presented through forest plots. Additionally, a sensitivity analysis was conducted to assess the impact of potential bias in some included RCTs on the study findings, and studies with factors contributing to heterogeneity were excluded. Moreover, in cases where *I*^2^ > 50%, subgroup analyses were performed to investigate potential factors that may contribute to the heterogeneity of the primary outcomes. These factors included age, duration of intervention, and BMI. The purpose of conducting these analyses was to minimize the impact of population baseline information and cumulative effects of interventions on the heterogeneity of results. Furthermore, the meta-regression analyses took into account participants' characteristics such as age, BMI, LVEF, and peak VO_2_ at baseline, as well as intervention characteristics such as duration, in order to explain potential sources of heterogeneity. Egger's test was used to assess the potential publication bias of these results, with *p* < 0.1 indicating significant publication bias. When ≥10 studies were included in the meta-analysis, we used the funnel plot (Supplementary Table S2). The analyses were performed using STATA software (Stata/MP 17.0).

## 3 Results

### 3.1 Study selection

**Figure 1:**
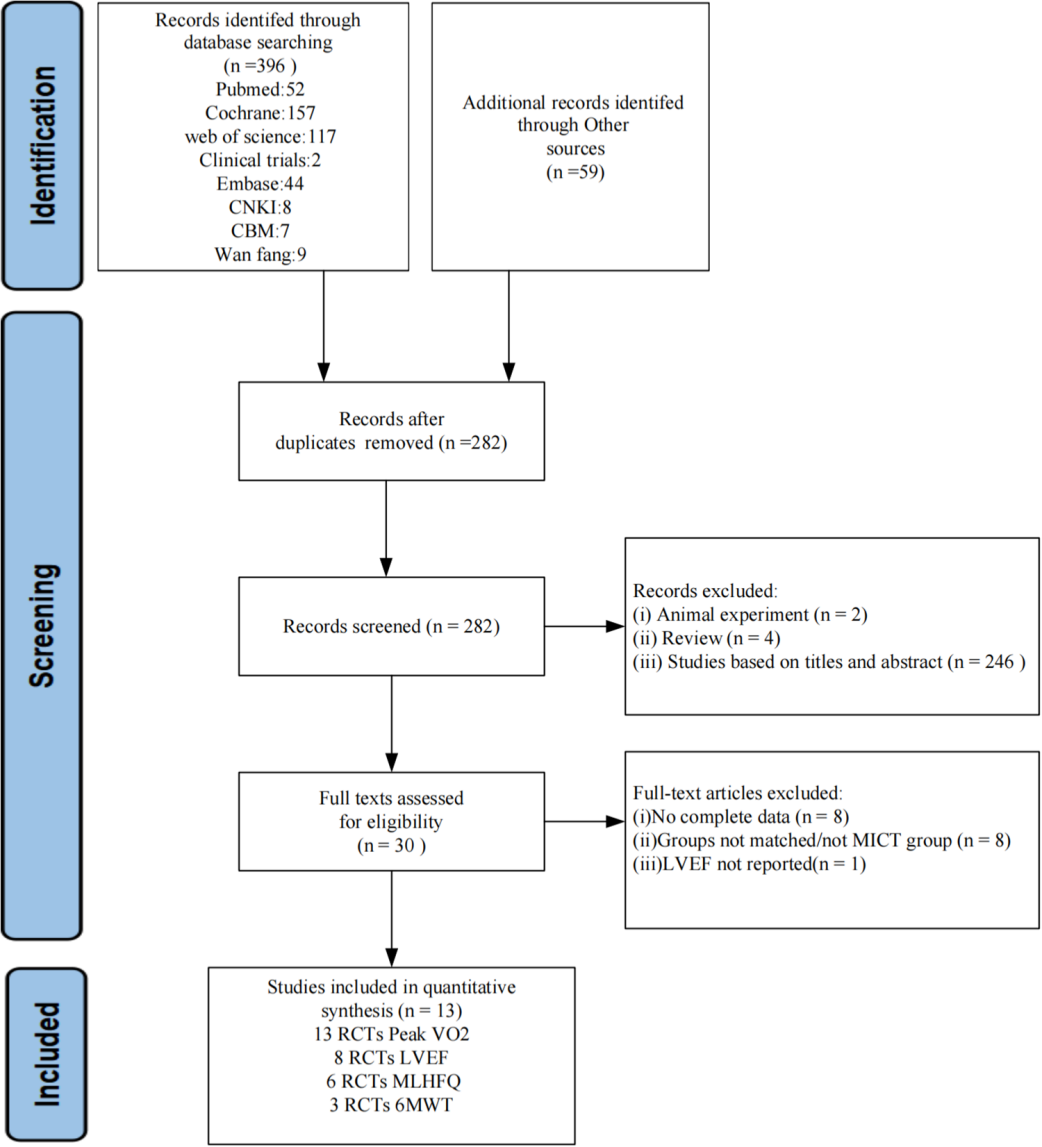
Flow chart of the study search selection according to PRISMA.

A total of 455 articles were obtained by searching 8 databases and conducting manual searches. After removing duplicates, 282 articles remained. Following a thorough evaluation of the title and abstract, 30 articles were identified as potentially meeting the criteria. Subsequently, we read the full text to further exclude 17 articles, 8 did not have complete data, 8 controls were not in the MICT group, and 1 did not report LVEF. In the end, a total of 13 randomized controlled trials (RCTs) were deemed eligible for inclusion. A flowchart summarizing the study selection process is shown in Figure 1.

### 3.2 Study characteristics

A total of thirteen studies (23–35) were conducted on patients with reduced ejection fraction in HF, with a total of 513 participants, of whom 454 (88%) were male. Among the participants, 262 underwent HIIT training, while 251 completed MICT training. There was an average age of 63 years and a BMI of 27 kg/m^2^ among the participants. The mean LVEF at baseline was 34%, and the study lasted from 3.5 weeks to 6 months. Based on all the studies included in the analysis, Peak VO_2_ was assessed using the cardiopulmonary exercise testing (CPET). The primary exercise modalities employed were cycling and uphill treadmill walking, and the exercise intensity was predominantly measured as a percentage of peak power or peak heart rate. However, two studies (30, 31) utilized a percentage of heart rate reserve (HRR), while one study (32) employed a percentage of Peak VO_2_. Table 1 shows the baseline demographic and clinical characteristics of the study participants. Table 2 summarizes the exercise training protocols for the HIIT and MICT groups.

**Table 1:** Characteristics of included studies.

Study	HIIT					MICT				
	Gender (M;F)	Age (years)	BMI (kg/m^2^)	LVEF (%)	Sample size	Gender (M;F)	Age (years)	BMI (kg/m^2^)	LVEF (%)	Sample size
Hornikx et al. (23)	5;5	64 ± 8	26 ± 4	30 ± 14	10	6;4	58 ± 11	29 ± 4	31 ± 14	10
Papathanasiou et al. (24)	35;25	63.65 ± 6.71	27.9	35.88 ± 2.3	60	35;25	63.82 ± 6.71	27.3	36.03 ± 2	60
Besnier et al. (25)	11;5	59 ± 13	25 ± 5	36 ± 8	16	11;4	59.5 ± 12	28 ± 5	36 ± 7	16
Ellingsen et al. (26)	63;14	65 ± 22.4	27.6 ± 5.4	29 ± 11.19	77	53;12	60 ± 14.4	27.5 ± 6.4	29 ± 12.4	65
Ulbrich et al. (27)	15;0	53.15 ± 7.0	29.73 ± 5.4	35.40 ± 6.7	15	12;0	54.02 ± 9.9	27.47 ± 4.6	32.8 ± 7.7	12
Benda et al. (28)	9;1	63 ± 8	28.1 ± 7.5	37 ± 6	10	10;0	64 ± 8	28.9 ± 4.7	38 ± 6	10
Koufaki et al. (29)	14;2	59.8 ± 7.4	28.9 ± 4.7	41.7 ± 10.3	16	13;4	59.7 ± 10.8	29.5 ± 4.7	35.2 ± 6.4	17
Iellamo et al. (30)	16;2	67.2 ± 6	28.3 ± 3	34.1 ± 6	18	15;3	68.4 ± 8	28.1 ± 2	35.6 ± 7	18
Iellamo et al. (31)	10;0	62.2 ± 8	27.8 ± 2	33.7 ± 4.79	10	10;0	62.6 ± 9	27.2 ± 3	31.5 ± 6.9	10
Fu et al. (32)	10;5	67.5 ± 1.8	24.6	38.3 ± 3.5	15	9;6	66.3 ± 2.1	24.5	38.6 ± 4.8	15
Freyssin et al. (33)	6;6	54 ± 9	24.8 ± 4.0	27.8 ± 4.7	12	7;7	55 ± 12	24.1 ± 5.4	30.7 ± 7.8	14
Wisloff et al. (34)	7;2	76.5 ± 9	24.5 ± 3	28.0 ± 7.3	9	7;2	74.4 ± 12	24.7 ± 3	32.8 ± 4.8	9
Myers et al. (35)	9;1	59.2 ± 12.2	26.5 ± 4.1	34.5 ± 10.5	10	14;0	61.5 ± 7.1	27.2 ± 4.2	30.7 ± 10.3	14

**Table 2:** Characteristics for HIIT and MIICT interventions.

Study	Type exercise	Frequency (days/week)	Intensity (% max) (interval; rest)	Time per week (min)	Exercise modality	Length (weeks)	Attendance rate, dropouts, adverse events
Hornikx et al. (23)	HIIT	3	5 × 3 min 80% Wpeak; 4 × 3 min 40% Wpeak	117	Running or cycle	12	Attendance: NR; dropouts = 1 (10%); adverse = 3 (33%)
	MICT		50% Wpeak	180			Attendance: NR; dropouts = 1 (10%); adverse = 1 (10%)
Papathanasiou et al. (24)	HIIT	2	3 × 90% HRmax; 2 × 70% HRmax	80	Upper and lower limb movements; Flexibility exercises.	12	Attendance = 100%; dropouts = 0; adverse = 0
	MICT		70% HRmax		Cycle		Attendance = 100%; dropouts = 0; adverse = 0
Besnier et al. (25)	HIIT	5	2 × 8 min 100% PPO (30 s traning, 30 s rest)	150	Cycle	3.5	Attendance: 100%; Dropouts = 0; adverse = 0
	MICT		60% PPO	200			Attendance: 94%; Dropouts = 1; adverse = 0
Ellingsen et al. (26)	HIIT	3	4 × 4 min 90–95% HRmax; 3 × 3 min 60–70% HRmax	114	Running or cycle	12	Attendance = 94%; dropouts = 5; adverse = 9
	MICT		60%–70% HRmax	141			Attendance = 89%; dropouts = 8; adverse = 6
Ulbrich et al. (27)	HIIT	3	(4∼6) × 3 min 95% HRmax; 3 min 70% HRmax	180	Uphill treadmill walking or running	12	Attendance = 83%; dropouts = 3; adverse = NR
	MICT		75% HRmax				Attendance = 80%; dropouts = 2; adverse = NR
Benda et al. (28)	HIIT	2	10 × 1 min 90% Maximal workload; 10 × 2.5 min 30% Maximal workload	90	Cycle	12	Attendance NR; dropouts = 2; adverse: NR
	MICT		60%–75% maximal workload	100			Attendance NR; dropouts = 2; adverse: NR
Koufaki et al. (29)	HIIT	3	30 s 100% PPO; 1 min 30% PPO	90	Cycle	24	Attendance = 94%; dropouts = 1; adverse = 1
	MICT		40%–60% VO_2_peak	120			Attendance = 100%; dropouts = 3; adverse = 3
Iellamo et al. (30)	HIIT	3	4 × 4min 80%–85% HRR; 3 × 3min 45%–50% HRR	135	Uphill treadmill walking	12	Attendance = 88%; dropouts = 1; adverse = 0
	MICT		45%–60% HRR				Attendance = 84%; dropouts = 2; adverse = 0
Iellamo et al. (31)	HIIT	3	4 × 4 min 75%–80% HRR; 3 × 3min 45%–50% HRR	120	Uphill treadmill walking	12	Attendance = 100%; dropouts = 2; adverse = NR
	MICT		45%–60% HRR	135			Attendance = 100%; dropouts = 2; adverse = NR
Fu et al. (32)	HIIT	3	5 × 3 min 80% VO_2_peak (≈80% HRR); 4 × 3 min 40% VO_2_peak (≈40% HRR)	108	Cycle	12	Attendance = NR; dropouts = 1; adverse = NR
	MICT		60% VO_2_peak (≈60% HRR)				Attendance = NR; dropouts = 2; adverse = NR
Freyssin et al. (33)	HIIT	5	12 × 30 s 80% maximal power; 11 × 60 s rest	168	Cycle	8	Attendance = 100%; dropouts = 0; adverse = 0
	MICT		VT1 heart rate	360			Attendance = 100%; dropouts = 0; adverse = 0
Wisloff et al. (34)	HIIT	3	4 × 4 min 90%–95% HRpeak; 3 × 3 min 50%–70% HRpeak	114	Uphill treadmill walking	12	Attendance = 92%; dropouts = 0; adverse = 0
	MICT		70%–75% HRpeak	141			Attendance = 95%; dropouts = 1; adverse = 0
Myers et al. (35)	HIIT	3	100% WRpeak (30 s traning, 30 s rest)	120	Cycle	12	Attendance = NR; dropouts = 4; adverse = NR
	MICT		50%–65% WRpeak				Attendance = NR; dropouts = 1; adverse = NR

### 3.3 Quality and bias assessment in the included studies

**Figure 2:**
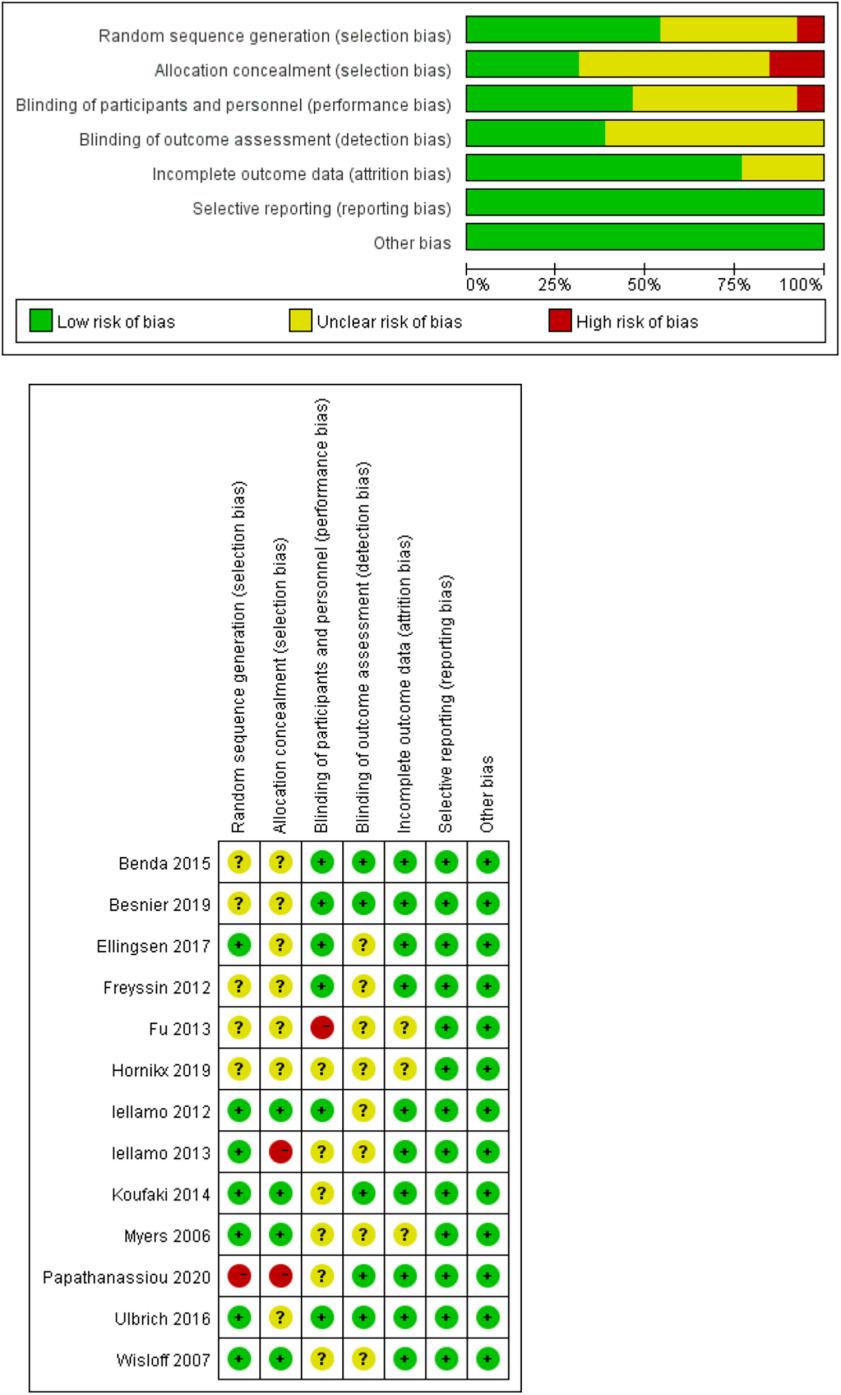
Risk of bias assessment.

It is worth noting that all studies included in the analysis used randomization. However, only 7 (54%) of these studies provided a description of the method used for random sequence generation. Additionally, 4 (7.5%) studies reported allocation concealment, 12 (92%) studies reported blinding of participants. Notably, none of the studies employed blinding for the participants. Moreover, 5 (38%) studies reported blinding of outcome measures, 3 (23%) studies had unclear completeness of outcome data, and none of the studies reported data selectively (Figure 2).

### 3.4 Quality of meta-analysis evidence

The detailed results of the GRADE assessment are presented in Table 3, Peak VO_2_, 6MWT, resting heart rate, and peak heart rate are determined to have low quality evidence. LVEF and MLHFQ, however, are determined to have moderate quality evidence.

**Table 3:** Results of the GRADE.

Quality assessment							No of patients		Outcome measures MD (95% CI)	Quality	Importance
No of studies	Design	Risk of bias	Inconsistency	Indirectness	Imprecision	Other considerations	HIIT	MICT			
Peak VO_2_ (better indicated by lower values)											
13	Randomised trials	Serious^a^	Serious^b^	No serious indirectness	No serious imprecision	None	262	251	1.78 (0.8 to 2.76)	⊕⊕OO Low	Critical
LVEF (better indicated by lower values)											
7	Randomised trials	Serious^a^	No serious inconsistency	No serious indirectness	No serious imprecision	None	146	129	3.84 (2.68 to 5.00)	⊕⊕⊕O Moderate	Important
6MWT (better indicated by lower values)											
3	Randomised trials	Serious^a^	No serious inconsistency	No serious indirectness	Serious^c^	None	84	84	28.13 (14.56 to 41.7)	⊕⊕OO Low	Important
MLHFQ (better indicated by lower values)											
6	Randomised trials	Serious^a^	No serious inconsistency	No serious indirectness	No serious imprecision	None	113	111	−4.45(−6.25 to −2.64)	⊕⊕⊕O Moderate	Important
Resting heart rate (better indicated by lower values)											
5	Randomised trials	Serious^a^	No serious inconsistency	No serious indirectness	Serious^d^	None	124	112	−0.15 (−1.68 to 1.37)	⊕⊕⊕OO Low	Important
Peak heart rate (better indicated by lower values)											
7	Randomised trials	Serious^a^	No serious inconsistency	No serious indirectness	Serious^d^	None^d^	144	133	0.5 (−2.69 to 3.69)	⊕⊕OO Low	Important

^a^ Most studies had moderate bias in randomization, allocation concealment, and blinding.

^b^ *I*^2^ > 50%.

^c^ Small sample size of included studies.

^d^ Wide confidence intervals for the study.

### 3.5 Results of the meta-analysis

#### 3.5.1 Analysis of peak VO_2_

Thirteen studies (23–35) assessed the Peak VO_2_ as outcome.

**Figure 3:**
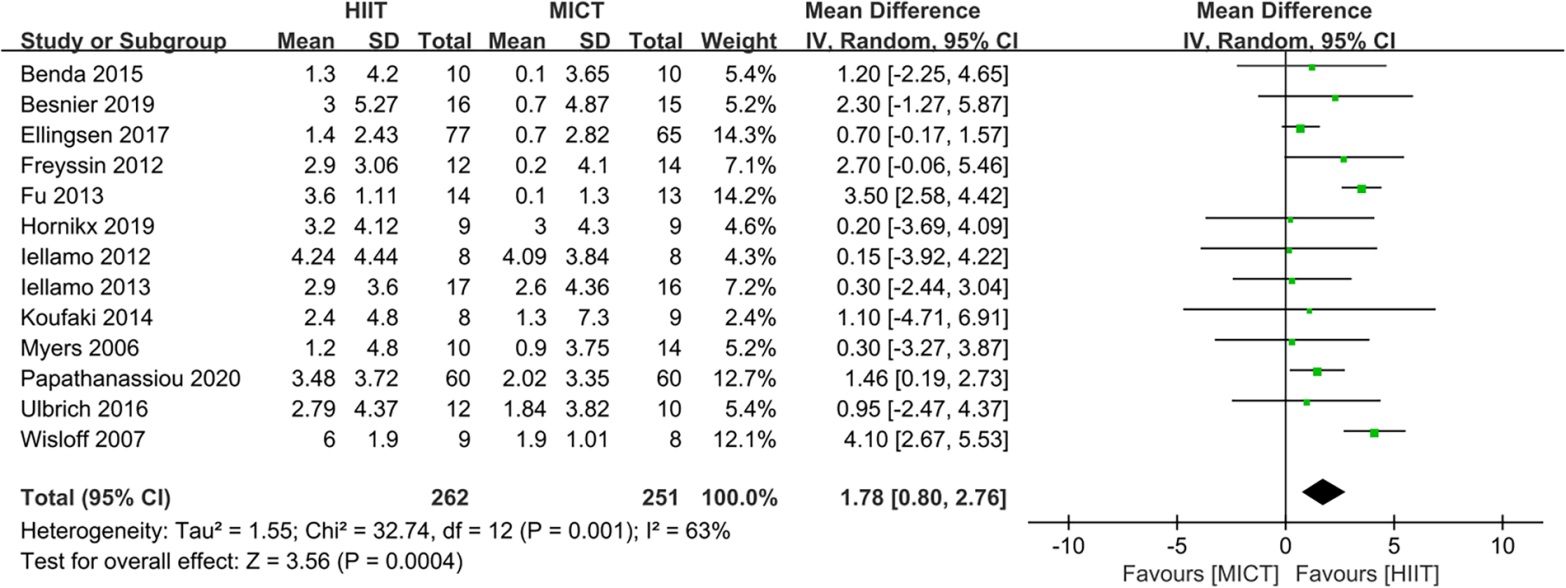
Forest plots of the effect of HIIT vs. MICT on peak VO_2_.

**Figure 4:**
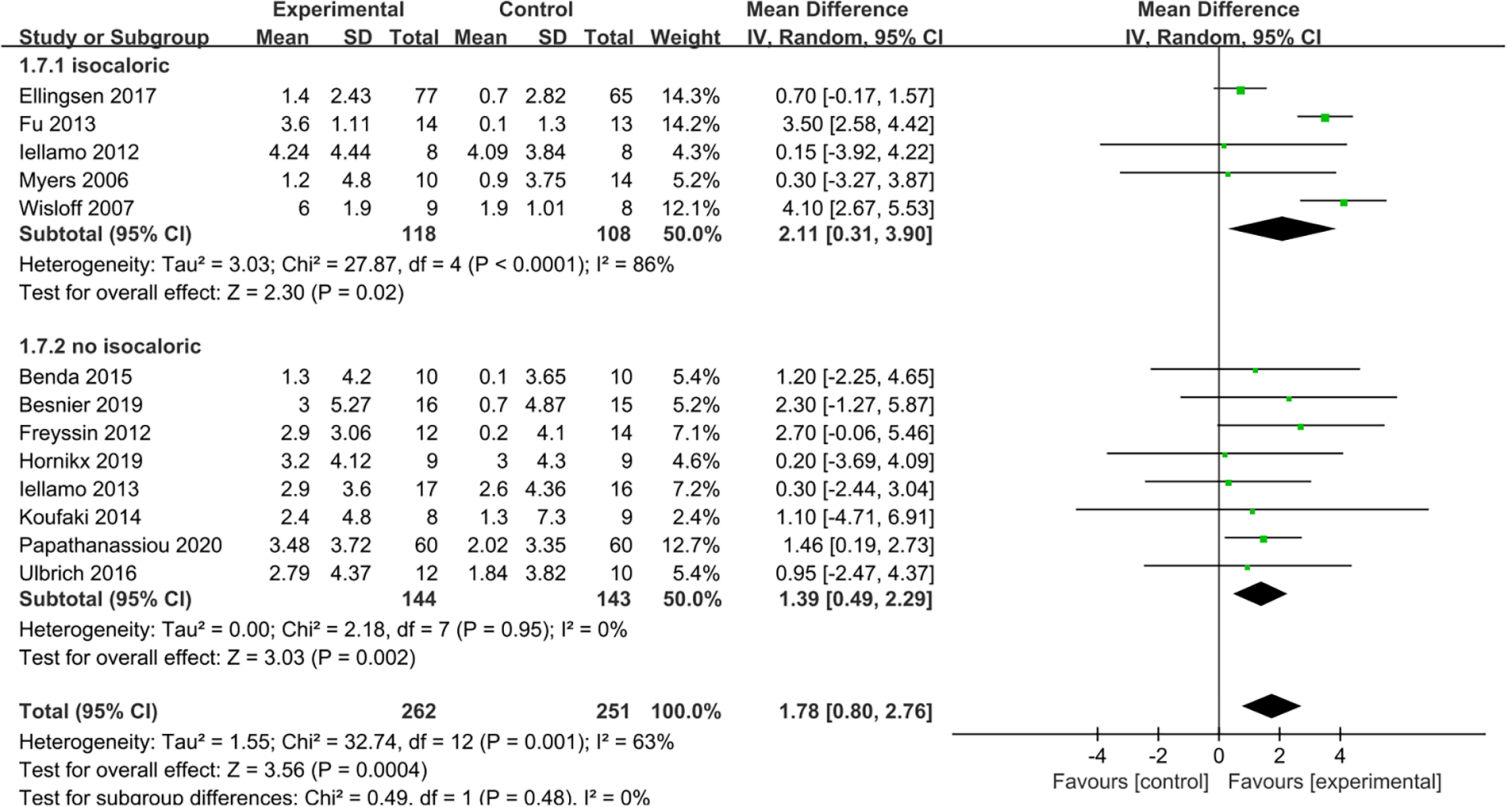
Subgroup analysis of HIIT vs. MICT on peak VO_2_ according to isocaloric or no isocaloric.

The results indicated that HIIT significantly increased the Peak VO_2_ compared to MICT (MD = 1.78, 95% CI for 0.80–2.76; 513 participants; 13 studies; *I*^2 ^= 63%; *P* = 0.0004; Figure 3). Only 5 of the studies (26, 31, 32, 34, 35) used an isocaloric exercise protocol, in contrast to 8 studies (23–25, 27–30, 33) that didn't report whether the HIIT and MICT protocols were isocaloric. Subgroup analyses demonstrated that HIIT significantly enhanced Peak VO2 compared to MICT in studies involving isocaloric exercise training (MD = 2.11, 95% CI for 0.31–3.90; 226 participants; 5 studies; *I*^2^ = 86%; *p* = 0.02) as well as studies not involving isocaloric exercise training (MD = 1.39, 95% CI for 0.49–2.29; 287 participants; 8 studies; *I*^2^ = 0%; *p* = 0.002) (Figure 4).

**Figure 5:**
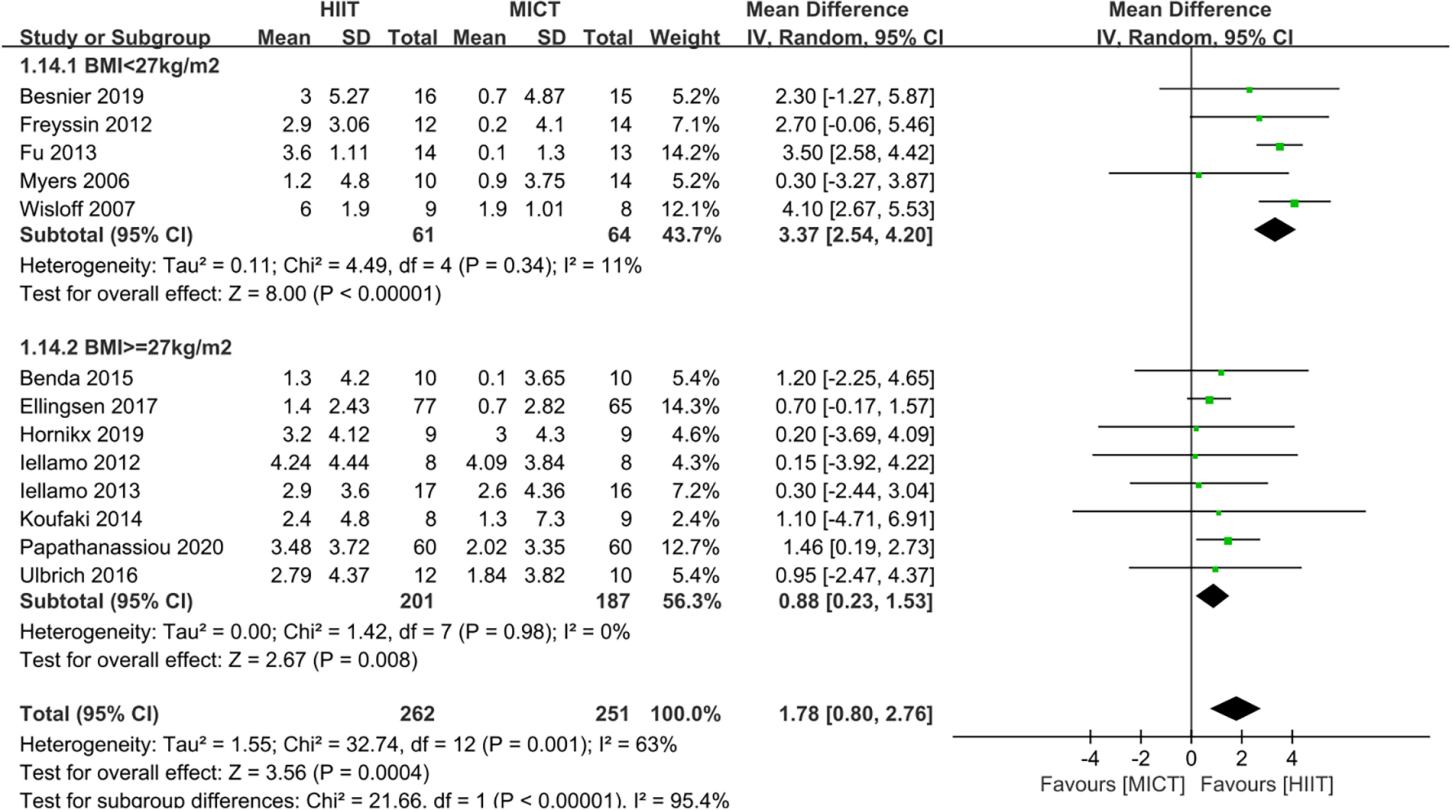
Subgroup analysis of HIIT vs. MICT on peak VO_2_ according to BMI. (<27 kg/m^2^, ≥27 kg/m^2^).

**Figure 6:**
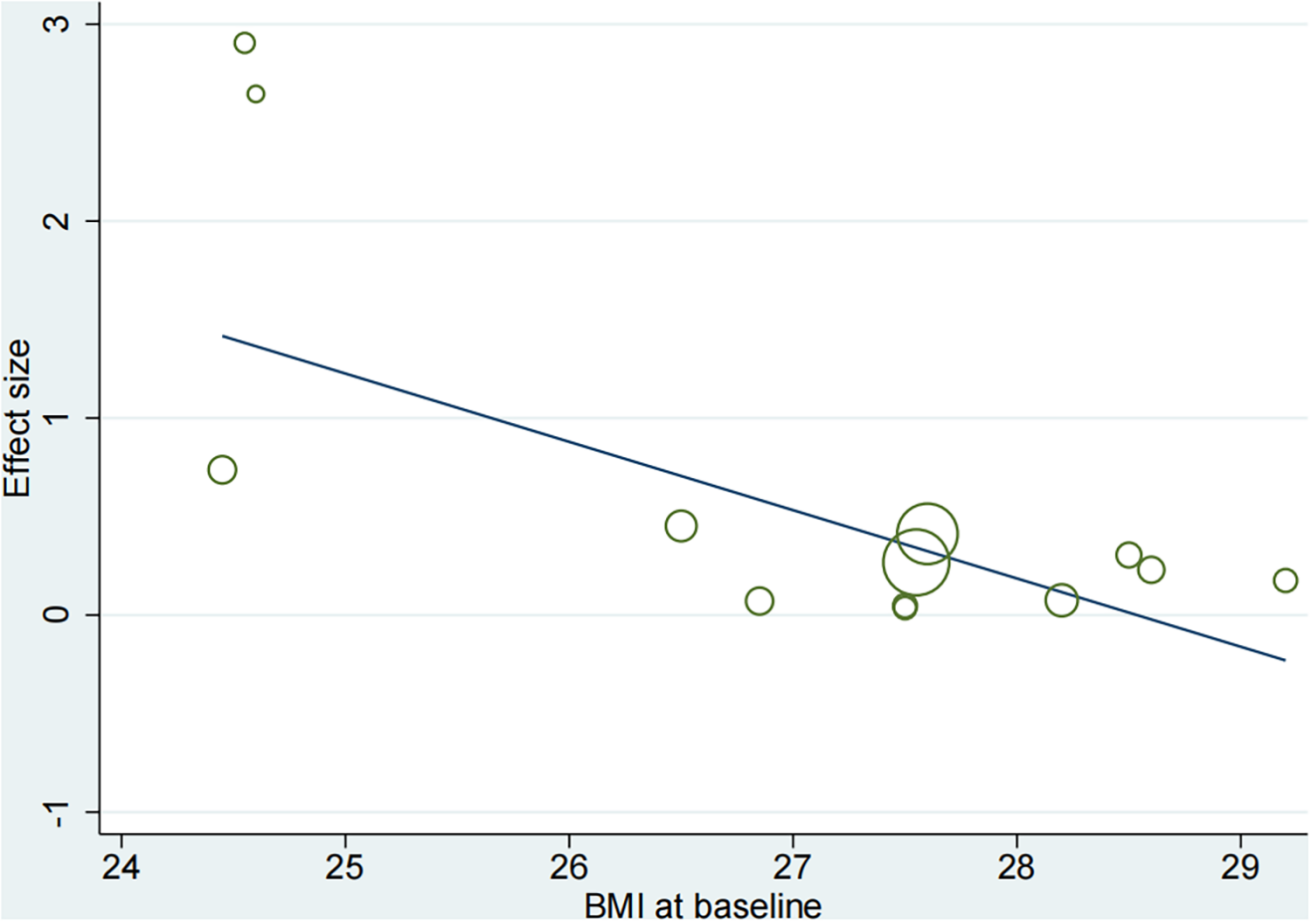
Meta-regression analysis of peak VO_2_ and BMI (body mass index).

Based on the mean age of the participants, subgroup analyses demonstrated that HIIT significantly improved the Peak VO_2_ compared to MICT in patients aged ≤ 60 years old (MD = 1.67, 95% CI for 0.09–3.24; 120 participants; 5 studies; *I*^2^ = 0%; *p* = 0.04) and patients aged > 60 years old (MD = 2.07, 95% CI: 1.56–2.57; participants = 393; 8 studies; *I*^2^ = 77%; *p* < 0.00001) (Supplementary Figure S1). Subgroup analyses according to the duration of intervention showed that HIIT substantially increased the Peak VO_2_ compared to MICT in both interventions lasting <12weeks (MD = 2.55, 95% CI for 0.37–4.73; 57 participants; 2 studies; *I*^2^ = 0%; *P* = 0.02) and those lasting ≥12 weeks (MD = 1.65, 95% CI for 0.55–2.75; 456 participants; 11 studies; *I*^2^ = 69%; *P* = 0.003) (Supplementary Figure S2). In subgroup analyses considering BMI, the meta-analyses indicated that HIIT led to a significant increase in Peak VO_2_ compared to MICT in patients with BMI <27 kg/m^2^ (MD = 3.37, 95% CI for 2.54–4.20; 125 participants; 5 studies; *I*^2^ = 11%; *P* < 0.00001) and patients with BMI ≥27 kg/m^2^ (MD = 0.88, 95% CI for 0.23–1.53; 388 participants; 8 studies; *I*^2^ = 0%; *P* = 0.008; Figure 5). The BMI of the study participants was determined to be the source of heterogeneity based on the subgroup analyses described above. Furthermore, meta-regressions were conducted to examine the relationship between the difference in peak VO_2_ and the background factors of the patients. There was no significant correlation observed between post-intervention differences in peak VO_2_ and age (≤60 years or >60 years), LVEF (<35% or ≥35%), duration of intervention (<12 weeks or ≥12 weeks), or peak VO_2_ at baseline (<16 ml/kg/min or ≥16 ml/kg/min). However, the difference in peak VO_2_ between HIIT and MICT showed a negative correlation with BMI (<27 kg/m^2^ or ≥27 kg/m^2^) (*r* = −0.57; *p* = 0.002; 95% CI: −0.86 to −0.29), (Table 4, Figure 6), which further validates the results of the subgroup analysis.

**Table 4:** Meta-regression analyses with peak VO_2_.

Factors	Coefficient	Standard error	*t*	*P*	95% CI	
Age	0.048	0.025	1.91	0.098	−0.012	0.108
BMI	−0.573	0.121	−4.71	0.002	−0.860	−0.285
LVEF	0.055	0.028	1.90	0.100	−0.014	0.123
Duration of intervention	0.057	0.034	1.64	0.146	−0.025	0.138
PeakVO_2_ at baseline	0.079	0.046	1.69	0.135	−0.032	0.189

#### 3.5.2 Analysis of left ventricular ejection fraction

**Figure 7:**
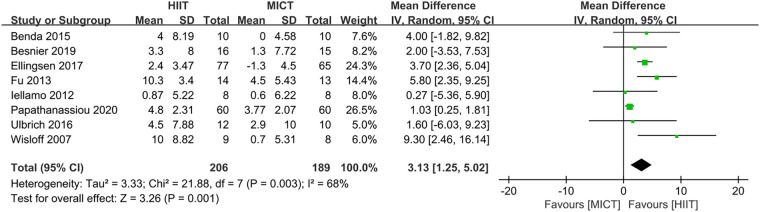
Forest plots of the effect of HIIT vs. MICT on LVEF.

**Figure 8:**
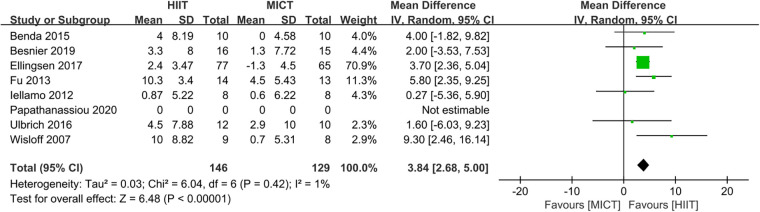
Sensitivity analysis of HIIT vs. MICT on LVEF.

Eight studies (24–28, 31, 32, 34) evaluated the LVEF as outcome. In comparison to MICT, HIIT demonstrated a significant increase in LVEF (MD = 3.13, 95% CI for 1.25–5.02; 395 participants; 8 studies; *I*^2^ = 68%; *P* = 0.001; Figure 7). A sensitivity analysis was conducted, suggesting that the study conducted by Papathanasiou et al. (24) may be the source of heterogeneity. Heterogeneity was significantly decreased by excluding this study (MD = 3.84, 95% CI for 2.68–5.00; 275 participants; 7 studies; *I*^2^ = 1%; *P* < 0.00001; Figure 8).

#### 3.5.3 Analysis of six-minute walk test

**Figure 9:**
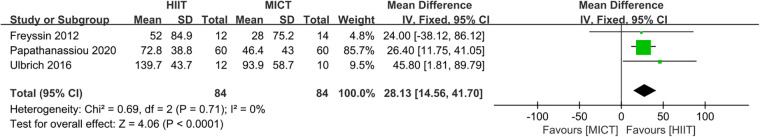
Forest plots of the effect of HIIT vs. MICT on 6MWT.

Three studies (24, 27, 33) assessed the 6MWT as outcome. HIIT led to significantly greater improvements in 6MWT compared to MICT (MD = 28.13, 95% CI for 14.56–41.70; 168 participants; 3 studies; *I*^2^ = 0%; *P* < 0.0001; Figure 9).

#### 3.5.4 Analysis of Minnesota living with heart failure questionnaire

**Figure 10:**
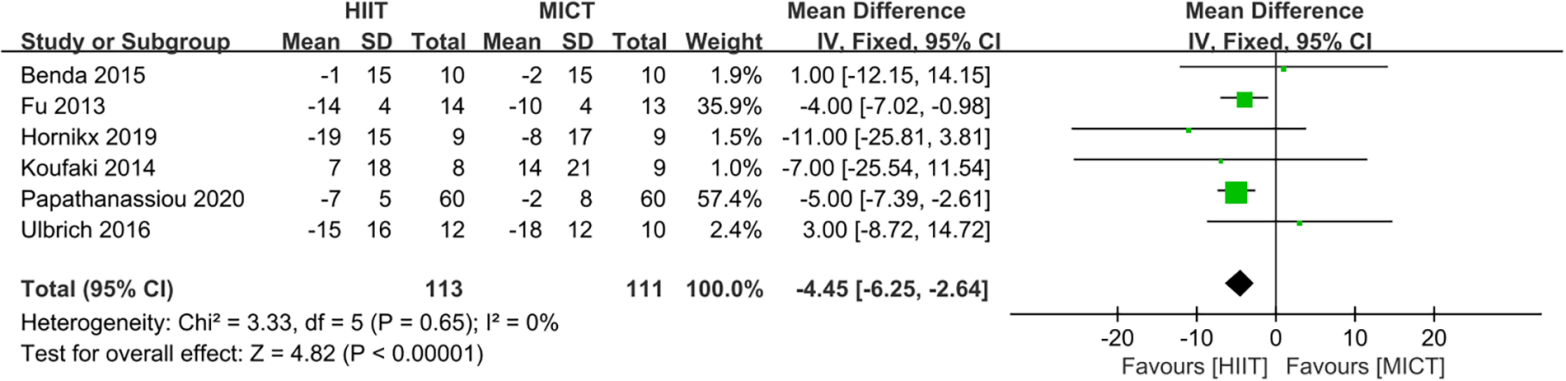
Forest plots of the effect of HIIT vs. MICT on MLHFQ.

Six studies (23, 24, 27–29, 32) evaluated the MLHFQ as a measure of QoL. HIIT was superior to MICT for improving MLHFQ. (MD = −4.45, 95% CI for −6.25 to −2.64; 224 participants; 6 studies; *I*^2^ = 0%; *P* < 0.00001; Figure 10).

#### 3.5.5 Analysis of resting heart rate and peak heart rate

**Figure 11:**
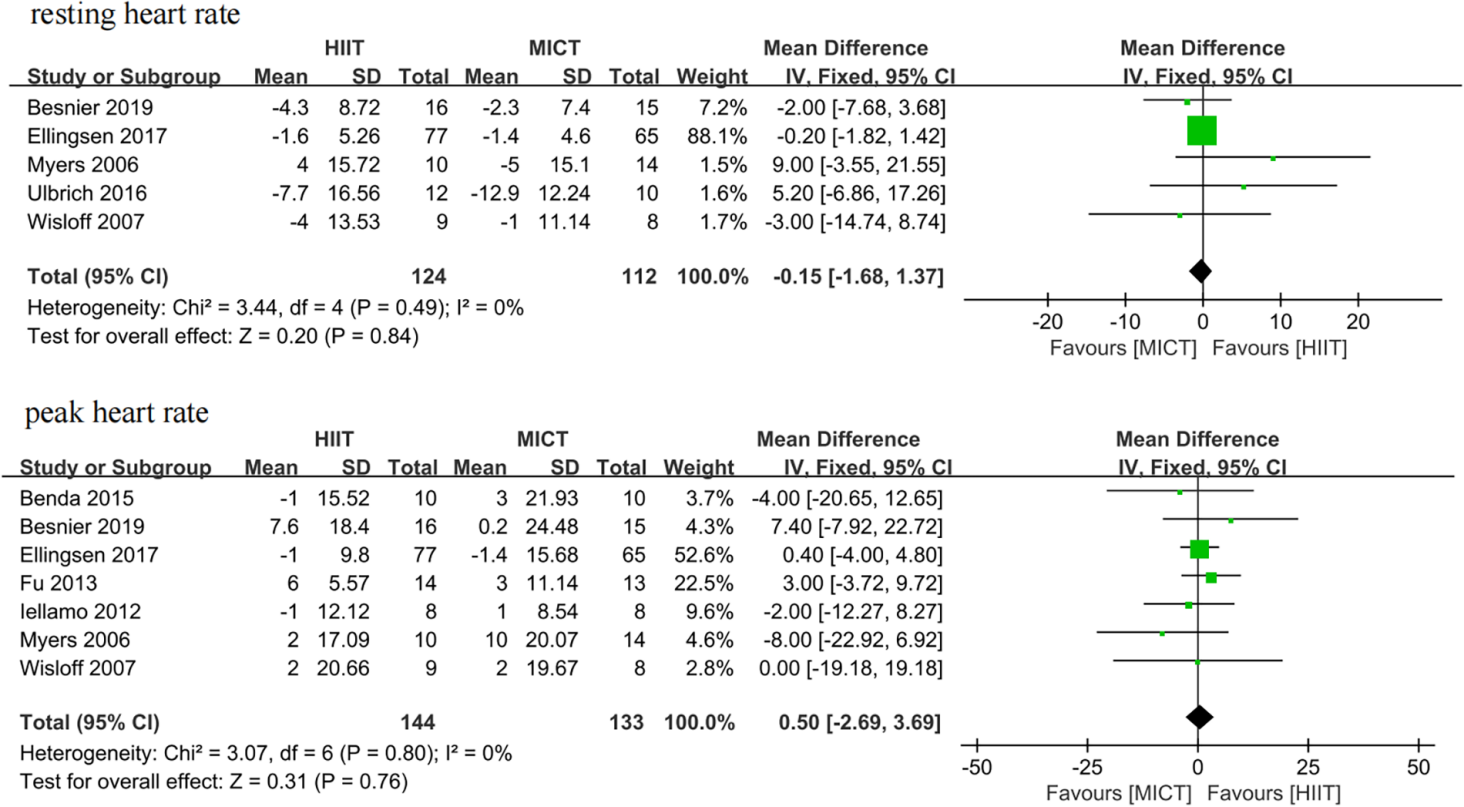
Forest plots of the effect of HIIT vs. MICT on resting heart rate and peak heart rate.

Five studies (25–27, 34, 35) and seven studies (25, 26, 28, 31, 32, 34, 35) assessed resting heart rate and peak heart rate as outcomes, respectively. The meta-analyses suggested that there was no significant difference in resting heart rate (MD = −0.15, 95% CI for −1.68 to 1.37; 236 participants; 5 studies; *I*^2^ = 0%; *P* = 0.84; Figure 11) and peak heart rate (MD = 0.50, 95% CI for −2.69 to 3.69; 277 participants; 7 studies; *I*^2^ = 0%; *P* = 0.76; Figure 11) between the HIIT and MICT groups.

## 4 Discussion

The objective of this systematic review and meta-analysis was to compare the effects of HIIT and MICT on aerobic and functional capacity in patients with HFrEF. The results demonstrated that HIIT was superior to MICT in terms of peak VO_2_, LVEF, 6MWT, and MLFHQ, while there were no significant differences in resting heart rate or peak heart rate. The findings of the current study's meta-analysis on peak VO_2_ are consistent with previous studies (12, 36). Additionally, when isocaloric protocols were analyzed in subgroups, high-intensity interval training remained superior in terms of peak VO_2_. In accordance with Okamura et al.' s previous study (36), additional meta-regression analyses demonstrated that the difference in peak VO_2_ was negatively correlated with BMI. However, Okamura et al.'s Meta-analysis included studies with participants with HF, including heart failure with preserved ejection fraction and HFrEF. Consequently, the strength of the current study is that all participants were exclusively HFrEF, thereby mitigating the potential influence of disease type on the outcomes. Additionally, the study demonstrated that HIIT was also significantly more favorable than MICT with respect to LVEF and MLHFQ. This finding is noteworthy as previous studies (12, 13, 36–38) did not report any significant differences between HIIT and MICT. Furthermore, the systematic review is the first to investigate the differences in 6MWT and heart rate between HIIT and MICT in patients with HFrEF.

It is important to highlight that peak VO_2_ serves as a predictive factor for readmission and mortality in HF patients. Furthermore, this research emphasizes the importance of enhancing peak VO_2_ through cardiac rehabilitation (29). Chou et al. (39) conducted an investigation into the mechanisms through which HIIT promotes the enhancement of peak VO_2_. Their findings revealed that HIIT improves platelet mitochondrial function, and there exists a positive correlation between whole-body aerobic capacity and platelet's mitochondrial reserve capacity. Additionally, peak VO_2_ serves as a vital indicator of aerobic capacity. The analysis of subgroups demonstrated that the difference in peak VO_2_ between HIIT and MICT was associated with BMI, but not with age, duration of the study, etc. Additional meta-regression analyses indicated a negative correlation between the difference in peak VO_2_ among the HIIT and MICT groups and BMI, suggesting that HIIT may be more effective in enhancing aerobic exercise capacity in HFrEF patients with a lower BMI. It has been reported that over half of HF patients, particularly those with HFrEF, suffer from skeletal sarcopenia, which has been linked to increased mortality rates in this population (40, 41). A low BMI is the primary characteristic of skeletal sarcopenia (42). In their study, Abshire et al. (43) found that a decreased BMI correlated with diminished bone density and an increased risk of osteoporosis among individuals with HF. Furthermore, it has been documented (44) that HIIT exhibits superior efficacy in reducing risk factors associated with skeletal sarcopenia and mitigating the morphological and functional deterioration linked to this condition, surpassing the benefits of resistance training. Notably, compared to the MICT group, the HIIT group experienced a significant reduction in BMI and a substantial increase in peak VO_2_, according to the study by Tadiotto et al. (45). Based on the results of the present meta-analysis, we therefore hypothesize that HIIT is preferable to MICT for increasing peak VO_2_ in HFrEF patients with a low. However, the limited number of studies investigating the impact of HIIT on aerobic capacity improvement in HF patients with a low BMI necessitates further high-quality research for validation.

The present study suggested that HIIT significantly increased LVEF in patients with HfrEF. Due to the significant heterogeneity observed, we conducted a sensitivity analysis and concluded that Papathanasiou et al.'s (16) study may have been a potential source of heterogeneity. We analyzed the possible reason why the study focused solely on patients with ischemic cardiomyopathy, hypertensive HF, and a limited number of patients with idiopathic dilated cardiomyopathy, rather than encompassing the entire range of HF conditions. It is important to note that HFrEF impacts cardiac hemodynamics and leads to a decrease in ventricular contraction, accompanied by compensatory remodeling of the left ventricle, which can temporarily compensate for abnormal hemodynamics and function but ultimately result in the worsening of cardiac function (46, 47). The LVEF serves as a crucial indicator of HF, displaying a direct correlation with mortality rate (48). The result of the present study is disagreement with Araújo's research (37), which may be attributed to the inclusion of only five randomized controlled studies and the uncertain risk of bias in most of the included studies.

In patients with chronic cardiovascular disease, the 6MWT is widely used to assess functional capacity and prognosis. The test effectively replicates activities of daily living and is particularly valuable for elderly patients, whose symptoms and activity performances are typically inferior to theoretical maximal exercise capacity (49, 50). In HF patients, the 6MWT has proven to be a dependable indicator of increased hospitalization rates and mortality, with distances less than 300 meters indicating a poor prognosis (51, 52). Additionally, the 6MWT can be used to estimate peak VO_2_ (53). Several RCTs (24, 27, 33) have demonstrated that patients with HF in the HIIT group experienced a significant increase in the six-minute walk distance (6MWD) compared to the MICT group. This finding suggests that HIIT is more effective in improving exercise endurance than MICT, which is consistent with the present study. However, it is important to note that only three studies exploring the effect of HIIT vs. MICT on 6MWD were included in the current meta-analysis, potentially indicating reporting bias. Further studies with larger sample sizes and longer follow-up periods will be necessary to accurately quantify the effects of HIIT on 6MWD.

The Minnesota Living with Heart Failure Questionnaire (MLHFQ) is a specialized self-assessment tool used to evaluate the QoL of individuals with HF, specifically assessing their symptoms and concerns (54). Research has indicated that HF patients who performed longer distances in the 6MWT also experience notable enhancements in MLHFQ scores (55). Furthermore, it has been suggested that improvements in QoL among HF patients are closely associated with advancements in exercise capacity and clinical symptoms (56). In a study conducted by Mansueto et al. (12), there was no statistically significant difference between HIIT and MICT in terms of improving QoL for patients with HF. This may be related to the large differences in the exercise protocols (intensity and duration, etc.) of the included studies, the significant heterogeneity found, and the fact that these come from very low-quality evidence. However, previous research (24, 27) has demonstrated that patients with HFrEF who underwent HIIT still exhibited significantly higher scores on the MLHFQ compared to those in the MICT group, both post-treatment and during the follow-up period, which is consistent with the present finding. The discrepancy between our results and Okamura et al. (36) may be attributed to the fact that this study exclusively included patients with HFrEF, while Okamura et al. included patients with both HFrEF and HF with preserved ejection fraction. The MLHFQ score may have been improved more significantly in patients with HFrEF when HIIT was used.

There is positive correlation between an elevation in resting heart rate and an increase in mortality due to cardiovascular disease (57), which also impacts the long-term prognosis of patients with HFrEF, with notably improved prognoses for individuals who experience a reduction in resting heart rate of 20 beats per minute following discharge (58). It has been demonstrated that the significance of appropriate elevations in heart rate during exercise training in maintaining sufficient cardiac output and meeting metabolic requirements in patients with HF (59). The relationship between resting heart rate, peak heart rate, peak VO_2_, and adverse events has been explored in previous studies (60). Besnier et al. (25) demonstrated that HIIT led to a significant decrease in resting heart rate and an increase in peak heart rate compared to the MICT group. However, Ulbrich et al. (27) reported contradictory results, showing that MICT significantly reduced resting heart rate in patients with HF, which may be related to the fact that the study was only focus on male participants and was limited to HF patients with a mean age of 53 years. According to the current study, resting heart rate and peak heart rate were not statistically different between the HIIT and MICT groups. It may be due to the fact that most studies included were conducted for less than 12 weeks, with one study (29) lasting 24 weeks. As a result, changes in heart rate may not be detected in these studies because of the short duration. For further validation, additional randomized controlled trials need to be conducted with large sample sizes and long follow-up periods.

The meta-analysis also has a few limitations, which should be mentioned: Firstly, it is important to note that the majority of RCTs included in the meta-analysis lasted less than 12 weeks, and only one study lasted 24 weeks. Therefore, it remains unclear whether HIIT is more effective than MICT in patients with HFrEF in the long term. Secondly, it is worth mentioning that the majority of HFrEF patients included in this meta-analysis were elderly men (88%), with an average age of 63 years. As a result, it is unknown whether the findings are applicable to elderly female patients with HFrEF. Finally, despite the fact that all included studies were randomized controlled trials, it was unfeasible to effectively blind both participants and outcome assessors in the exercise training intervention studies. Furthermore, to investigate isocaloric training protocols in HF patients comprehensively, more large-scale RCTs of high quality must be conducted.

## 5 Conclusion

The systematic review indicates that HIIT was superior to MICT in terms of Peak VO_2_, LVEF, 6MWT, and MLHFQ scores in patients with HFrEF. Additionally, the sub-analysis of isocaloric protocols on peak VO_2_ also confirmed the superiority. Moreover, HIIT may be more effective in promoting exercise capacity among HFrEF patients with a low BMI. However, we found no evidence to support significant differences in resting heart rate and peak heart rate between HIIT and MICT.

## Data Availability

The original contributions presented in the study are included in the article/**Supplementary Material**, further inquiries can be directed to the corresponding authors.
