# Supplementary material for: High intensity interval training vs. moderate intensity continuous training on aerobic capacity and functional capacity in patients with heart failure: a systematic review and meta-analysis
Source: Front Cardiovasc Med. 2024 Feb 21;11:1302109. doi: 10.3389/fcvm.2024.1302109 (PMC10915068; doi:10.3389/fcvm.2024.1302109)

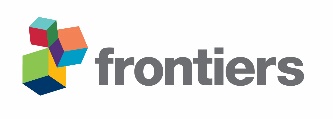
 Supplementary Material

# Supplementary Table S1. Searching strategy.

| Database | Search strategy |
| --- | --- |
| Pubmed  Web of Science | (((randomized controlled trial[Publication Type]) OR (randomized[Title/Abstract])) OR (placebo[Title/Abstract])) AND (((randomized controlled trial[Publication Type]) OR (randomized[Title/Abstract])) OR (placebo[Title/Abstract]) AND (((((((((((((((("Heart Failure"[Mesh]) OR (Heart Failure[Title/Abstract])) OR (Cardiac  Failure[Title/Abstract])) OR (Heart Decompensation[Title/Abstract])) OR (Decompensation, Heart[Title/Abstract])) OR (Heart Failure, Right-Sided[Title/Abstract])) OR (Heart Failure, Right Sided[Title/Abstract])) OR (Right-Sided Heart Failure[Title/Abstract])) OR (Right Sided Heart Failure[Title/Abstract])) OR (Myocardial  Failure[Title/Abstract])) OR (Congestive Heart Failure[Title/Abstract])) OR (Heart Failure, Congestive[Title/Abstract])) OR (Heart Failure, Left-Sided[Title/Abstract])) OR (Heart Failure, Left Sided[Title/Abstract])) OR (Left-Sided Heart Failure[Title/Abstract])) OR (Left Sided Heart Failure[Title/Abstract])) AND ((((((((((((("High-Intensity  Interval Training"[Mesh]) OR (High-Intensity Interval Training*[Title/Abstract])) OR (High Intensity Interval Training*[Title/Abstract])) OR (Interval Training,  High-Intensity[Title/Abstract])) OR (Interval Trainings, High-Intensity[Title/Abstract])) OR (Training, High-Intensity Interval[Title/Abstract])) OR (Trainings,  High-Intensity Interval[Title/Abstract])) OR (High-Intensity Intermittent Exercise[Title/Abstract])) OR (Exercise, High-Intensity Intermittent[Title/Abstract])) OR  (Exercises, High-Intensity Intermittent[Title/Abstract])) OR (High-Intensity Intermittent Exercises[Title/Abstract])) OR (Sprint Interval Training*[Title/Abstract])) OR (HIIT[Title/Abstract])))  #1 ：((((((((((((TS=(High-Intensity Interval Training*)) OR TS=(High Intensity Interval Training*))) OR TS=(Interval Training, High-Intensity)) OR TS=(Interval Trainings, High-Intensity)) OR TS=(Training, High-Intensity Interval)) OR TS=(Trainings, High-Intensity Interval)) OR TS=(High-Intensity Intermittent Exercise)) OR TS=(Exercise, High-Intensity Intermittent)) OR TS=(Exercises, High-Intensity Intermittent)) OR TS=(High-Intensity Intermittent Exercises)) OR TS=(Sprint Interval Training*)) OR  TS=(HIIT)  #2：((((((((((((((TS=(Heart Failure)) OR TS=(Cardiac Failure)) OR TS=(Heart Decompensation)) OR TS=(Decompensation, Heart)) OR TS=(Heart Failure, Right-Sided)) OR TS=(Heart Failure, Right Sided)) OR TS=(Right-Sided Heart Failure)) OR TS=(Right Sided Heart Failure)) OR TS=(Myocardial Failure)) OR TS=(Congestive Heart Failure)) OR TS=(Heart Failure, Congestive)) OR TS=(Heart Failure, Left-Sided)) OR TS=(Heart Failure, Left Sided)) OR TS=(Left-Sided Heart Failure)) OR TS=(Left Sided Heart Failure)  #3：(((TS=(randomized controlled trial)) OR TS=(randomized)) OR TS=(placebo)) OR TS=(random*)  #4：#1 AND #2 AND #3 |

| Database | Search strategy |
| --- | --- |
| Embase | #24 #2 OR #16 OR #17 OR #18 OR #19 OR #20 OR #21 OR #22 OR #23 OR #24 OR #25 OR #26  #23'heart failure, left-sided':ti,ab  #22'congestive heart failure':ti,ab  #21'myocardial failure':ti,ab  #20'heart failure, right-sided':ti,ab  #19'decompensation, heart':ti,ab  #18'heart decompensation':ti,ab  #17'cardiac failure':ti,ab  #16'heart failure':ti,ab  #15 #1 OR #3 OR #4 OR #5 OR #6 OR #7 OR #8 OR #9 OR #10 OR #11 OR #12 OR #13 OR #14  #14'hiit':ti,ab  #13'sprint interval training*':ti,ab  #12'high-intensity intermittent exercises':ti,ab  #11'exercises, high-intensity intermittent':ti,ab  #10'exercise, high-intensity intermittent':ti,ab  #9'high-intensity intermittent exercise':ti,ab  #8'trainings, high-intensity interval':ti,ab  #7'training, high-intensity interval':ti,ab  #6'interval trainings, high-intensity':ti,ab  #5'interval training, high-intensity':ti,ab  #4'high intensity interval training*':ti,ab  #3'high-intensity interval training*':ti,ab  #2'heart failure'/exp  #1'high intensity interval training'/exp |

| Database | Search strategy |
| --- | --- |
| Cochrane  Library | #1 MeSH descriptor: [Heart Failure] explode all trees  #2 (Heart Failure):ti,ab,kw OR (Cardiac Failure):ti,ab,kw OR (Heart Decompensation):ti,ab,kw OR (Decompensation, Heart):ti,ab,kw OR (Heart Failure, Right-Sided):ti,ab,kw  #3 (Right-Sided Heart Failure):ti,ab,kw OR (Myocardial Failure):ti,ab,kw OR (Congestive Heart Failure):ti,ab,kw OR (Heart Failure, Congestive):ti,ab,kw OR (Heart Failure, Left-Sided):ti,ab,kw  #4 (Left-Sided Heart Failure):ti,ab,kw OR (Left Sided Heart Failure):ti,ab,kw  #5 #1 OR #2 OR #3 OR #4  #6 MeSH descriptor: [High-Intensity Interval Training] explode all trees  #7 (High-Intensity Interval Training*):ti,ab,kw OR (High Intensity Interval Training*):ti,ab,kw OR (Interval Training, High-Intensity):ti,ab,kw OR (Interval Trainings, High-Intensity):ti,ab,kw OR (Training, High-Intensity Interval):ti,ab,kw  #8 (Trainings, High-Intensity Interval):ti,ab,kw OR (High-Intensity Intermittent Exercise):ti,ab,kw OR (Exercise, High-Intensity Intermittent):ti,ab,kw OR (Exercises, High-Intensity Intermittent):ti,ab,kw OR (High-Intensity Intermittent Exercises):ti,ab,kw  #9 (Sprint Interval Training*):ti,ab,kw OR (HIIT):ti,ab,kw  #10 #6 OR #7 OR #8 OR #9  #11 (randomized*):ti,ab,kw OR (placebo):ti,ab,kw  #12 #5 AND #10 AND #11  Cardiac Failure OR Heart Decompensation OR Myocardial Failure \| Studies With Results \| Interventional Studies \| Heart Failure \| High-Intensity Interval Training* OR High |
| clinical trials | Intensity Interval Training* OR Interval Training, High-Intensity OR Training, High-Intensity Interval OR High-Intensity Intermittent Exercise* OR Sprint Interval Training* |
| CNKI  Wanfang | (主题=高强度间歇训练 + 高强度间歇运动训练 + 高强度间歇性训练) AND (主题=心力衰竭 + 心衰)；  (主题:(高强度间歇训练 or 高强度间歇运动训练 or 高强度间歇性训练)and 主题:(心力衰竭 or 心衰)) |
| CBM | (("心力衰竭"[常用字段:智能] OR "心衰"[常用字段:智能]) AND ("高强度间歇训练"[常用字段:智能] OR "高强度间歇运动训练"[常用字段:智能] OR "高强度间歇性 训练"[常用字段:智能])) AND ("随机对照实验"[文献类型]) |

# Supplementary Table S2. Egger's test and funnel plot.

## PeakVO2


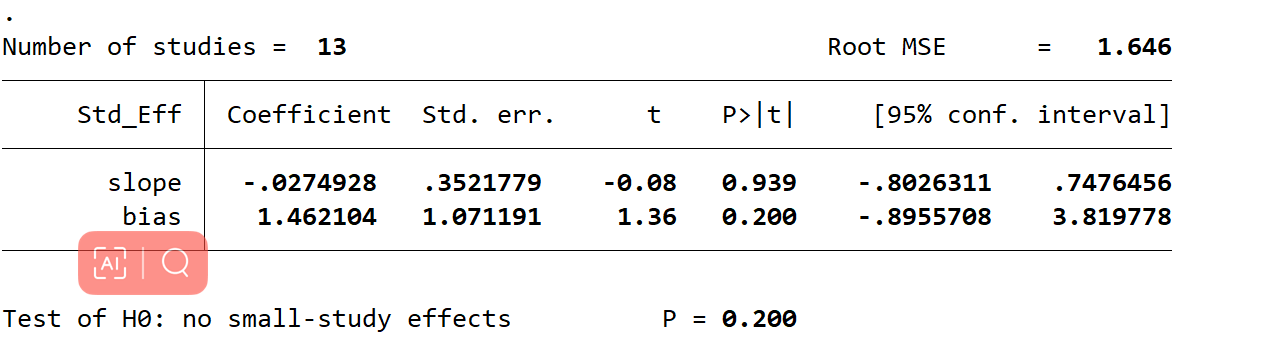


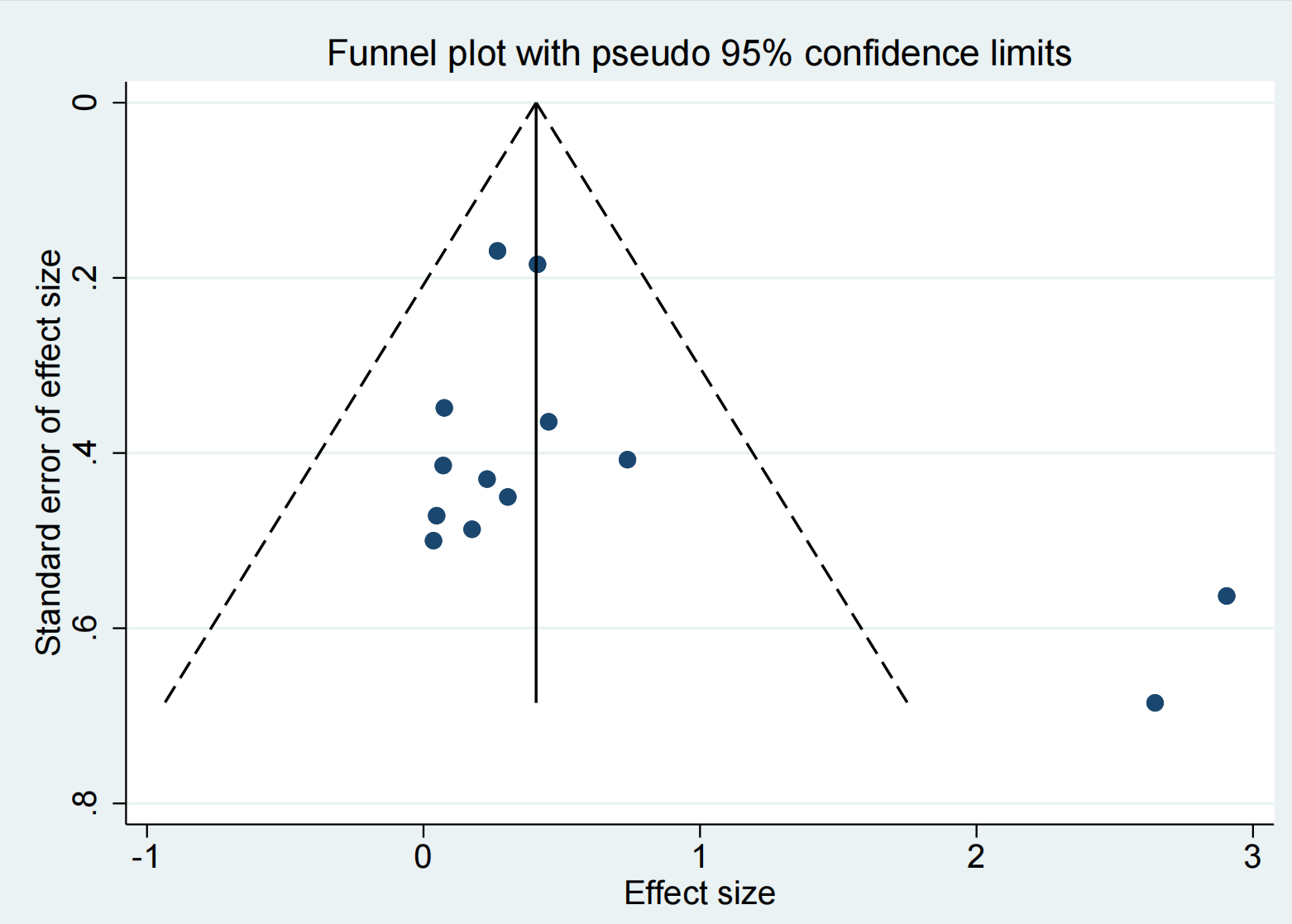


## LVEF


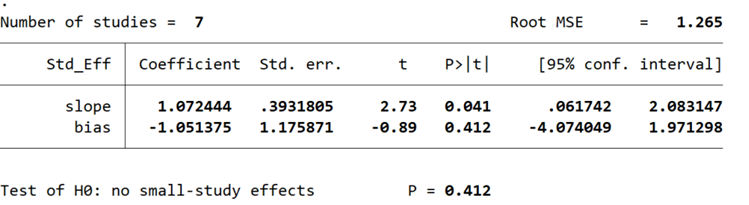


## 6MWT


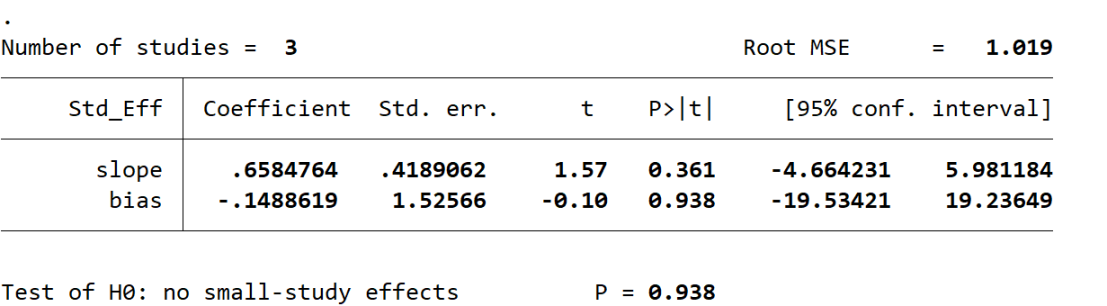


## MLHFQ


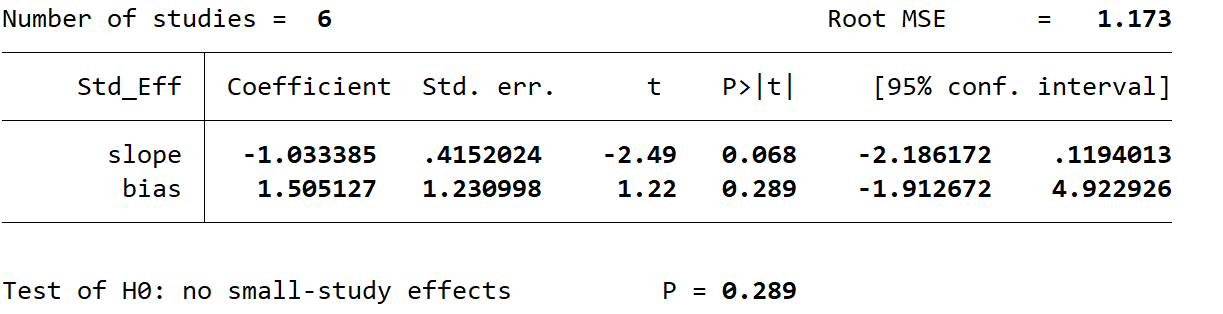


## Resting heart rate


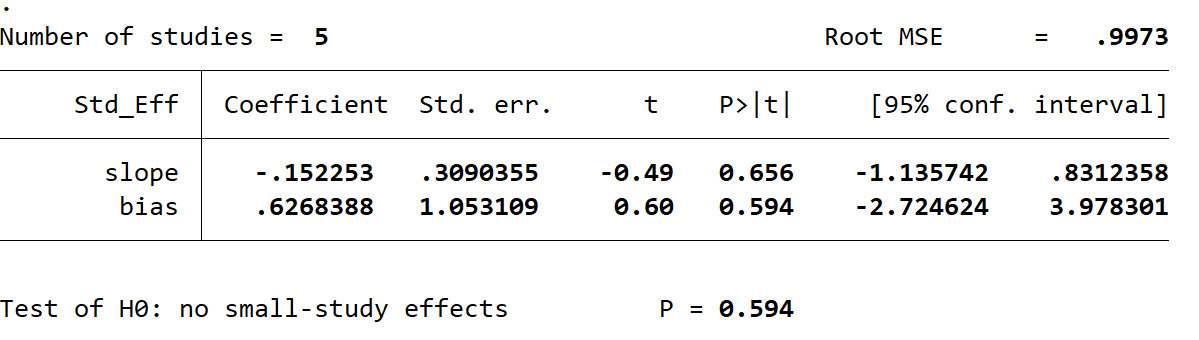


## Peak heart rate


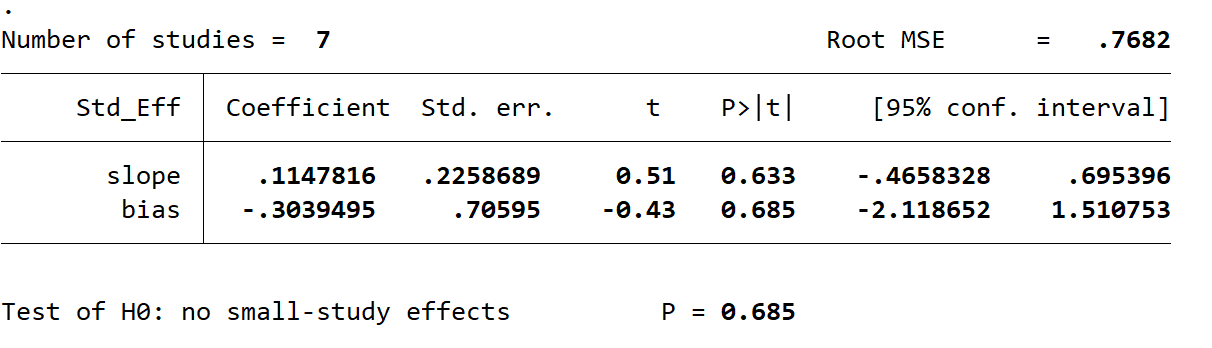


(P>0.1 indicates no significant publication bias.)

# **Supplementary Figure S1.** Subgroup analysis of HIIT versus MICT on peak VO2 according to the age of participants. (≤60 years old, > 60 years old)

**
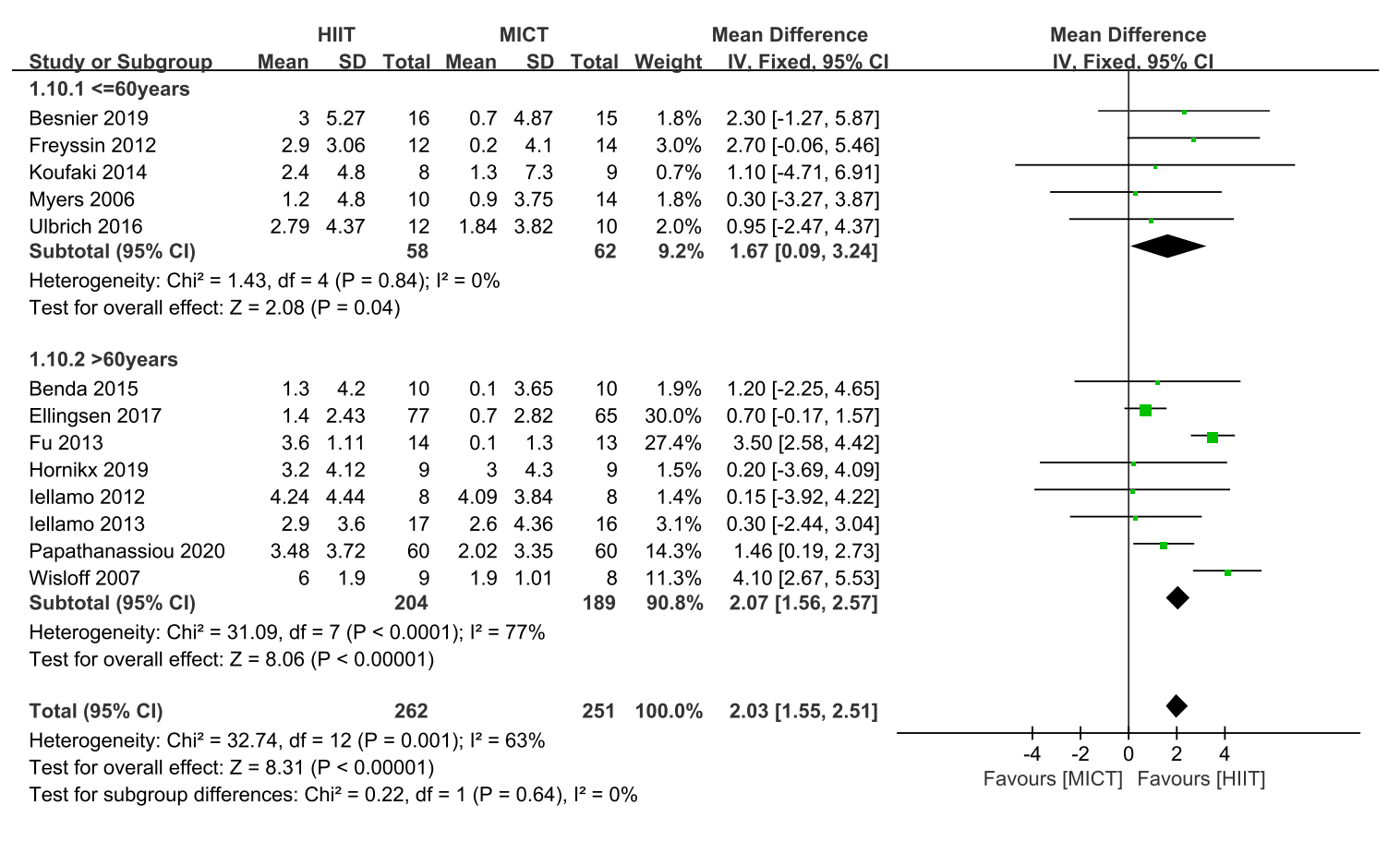
**

# Supplementary Figure S2. Subgroup analysis of HIIT versus MICT on peak VO2 according to the duration of intervention. (<12weeks, ≥12weeks)


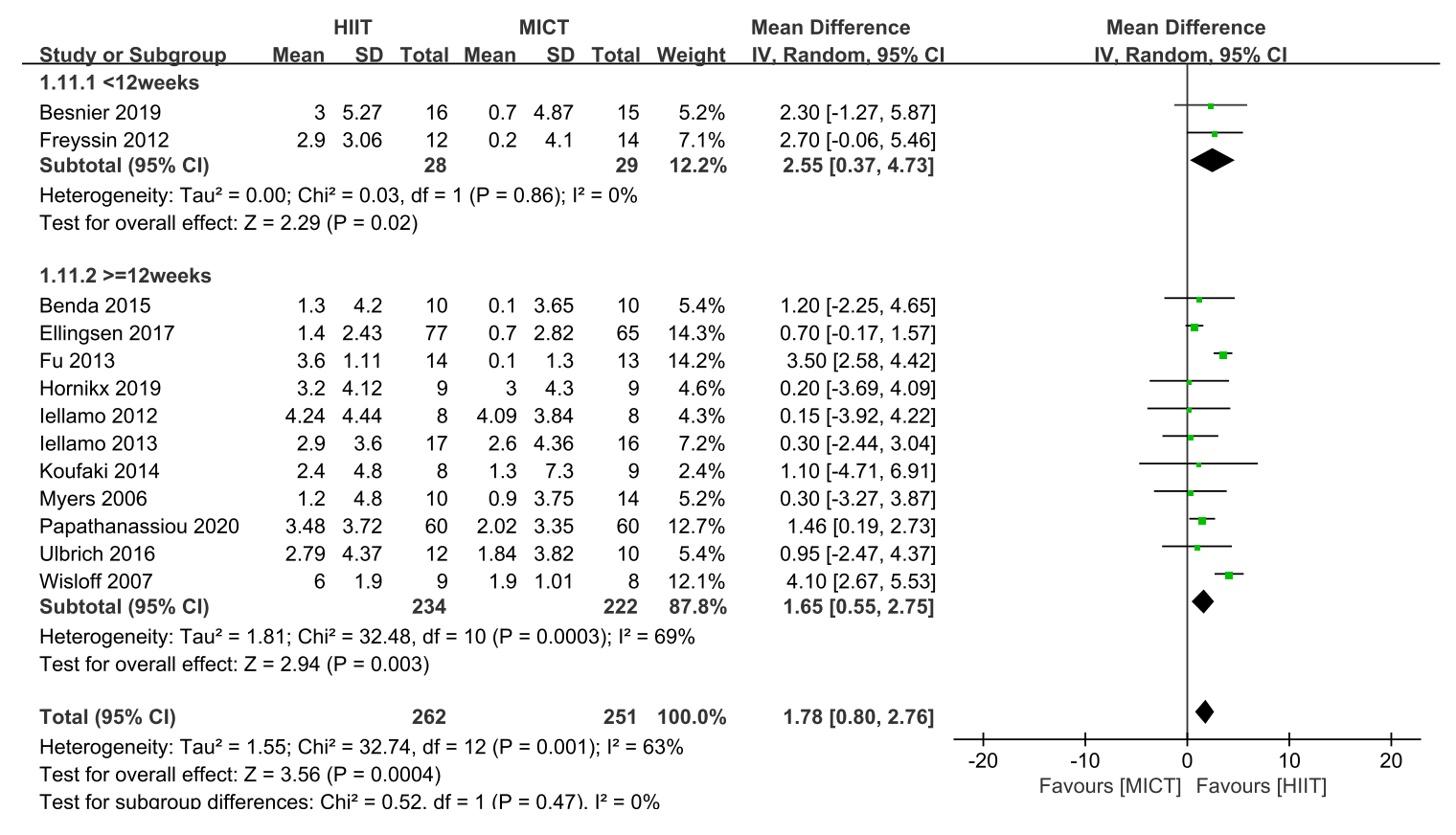

Supplement: Supplementary file 1 [file Datasheet1.docx]
